# Transcriptomics assisted by metabolomics analysis provides insights into regulation mechanisms during carcinogenic process in a hydrodynamically transfected liver cancer model

**DOI:** 10.1186/s12935-025-03937-1

**Published:** 2025-08-22

**Authors:** Qi Zhang, Yongfeng Li, Xiaojing Cheng, Zhouxiang Liao, Sha Wen, Xuejing Huang, Zhenyu Song, Min He, Lichao Yang

**Affiliations:** 1 Laboratory Animal Center, Guangxi Medical University, Nanning, 530021 China; 2 School of Public Health, Guangxi Medical University, Nanning, 530021 China; 3 State Key Laboratory of Targeting Oncology, Guangxi Medical University, Nanning, 530021 China; 4 Key Laboratory of Early Prevention and Treatment for Regional High Frequency Tumor (Guangxi Medical University), Ministry of Education, Nanning, 530021 China; 5 Guangxi Key Laboratory of Early Prevention and Treatment for Regional High Frequency Tumor, Nanning, 530021 China

## Abstract

**Background:**

Hepatocellular carcinoma (HCC) ranks among the most prevalent malignancies with a substantial mortality rate, and its pathogenesis is relatively complex. Animal models provide valuable tools for exploring the causes and mechanisms of malignancy. This study uses a novel hydrodynamic transfection mouse model to investigate the changes in key genes and metabolites during the development of HCC. By combining metabolomics-assisted transcriptomics assay, this research seeks to provide new insights into HCC development.

**Methods:**

A mouse model of HCC was established using hydrodynamic transfection coupled with the SB11 transposon system and the CRISPR-Cas9 system. C57BL/6J mice were used as experimental animals, with mice arbitrarily split into the control and experimental groups. The experimental group of mice underwent hydrodynamic transfection using mixed recombination carcinogenic plasmids that knocked out the tumor suppressor genes Pten and P53, while overexpressing the oncogenes β-catenin and c-Met. In contrast, the control group of mice was transfected with corresponding empty vectors. All mice were monitored for weight, activity, and blood routine examinations during the modeling phase. All mice were sacrificed upon completion of the modeling phase, and their liver specimens were harvested for pathological evaluations and metabolomics-assisted transcriptomics investigation.

**Results:**

In contrast to the control group, the experimental group mice exhibited notably smaller weight gain (*P* < 0.01) and markedly elevated serum ALT and AST levels (*P* < 0.001). At the end of the modeling period, visible white nodules appeared in the liver; hematoxylin and eosin (H&E) staining, immunohistochemistry, and electron microscopy revealed pathological features of HCC in the experimental group. Transcriptome analysis ascertained 2757 differentially expressed genes (DEGs) between HCC tissues and control liver tissues, with 2273 elevated and 484 diminished genes. KEGG pathway evaluation indicated substantial clustering of DEGs in cell cycle signaling pathways. Metabolome analysis showed the enrichment of differential metabolites in pathways related to ascorbate and alternate metabolism, choline metabolism in cancer, and glycerophospholipid metabolism. Notably, we observed significant differences in HCC progression between male and female mice after modeling, with female mice showing a higher incidence of HCC, greater liver-to-body weight ratios, and larger tumors than males. Transcriptome analysis and subsequent qRT-PCR demonstrated a significant downregulation of several glutathione transferase family genes (*Gata1*, *Gata2*, *Gstp1*, *Mgst1*) in the liver tissues of female mice versus males in the experimental group. Liver metabolome analysis revealed that female mice in the experimental group had 57 metabolites that differed from those of male mice, with 24 metabolites being upregulated and 33 downregulated. In particular, female mice exhibited markedly higher levels of glutamate, alanine, L-pyroglutamic acid, and glycerophospholipids (*P* < 0.05), while their pyridoxine levels were notably lower (*P* < 0.05) compared to male mice in the liver.

**Conclusions:**

In a hydrodynamic transfection-based mouse model of HCC, female mice showed higher tumor incidence, faster tumor growth, and more severe disease compared to male mice. This sex-based difference may be associated with lower hepatic expression of glutathione S-transferases (GSTs) in females. Targeting GST expression or activity may offer a potential strategy for sex-informed approaches to HCC prevention and therapy.

## Introduction

Hepatocellular carcinoma (HCC), ranking as the predominant hepatic malignancy globally, exhibits substantial morbidity and mortality rates alongside poor 5-year overall survival (OS) [1]. Several established causative elements for hepatic cancer encompass hepatitis B virus (HBV) infection, hepatitis C virus (HCV) infection, excessive alcohol consumption, cirrhotic liver disease, metabolic disorders, or various combinations thereof [2]. The progression of HCC occurs via sequential genetic and epigenetic modifications affecting proto-oncogenes and tumor suppressor genes within the hepatic microenvironment, where persistent liver injury leads to hepatic fibrosis or cirrhosis. However, the exact mechanisms underlying the signaling pathway perturbations and metabolite alterations triggered by oncogene activation and suppressor gene silencing during hepatoma carcinogenesis remain elusive. Consequently, gaining comprehensive insights into HCC pathogenesis is crucial for identifying effective therapeutic approaches for this disease.

Animal models of cancer provide an invaluable tool for exploring the causes and mechanisms of malignancy. These models serve as a crucial resource in cancer research, offering an alternative approach to studying the disease beyond human subjects. With advancements in cancer modeling, researchers can now observe and manipulate intricate disease processes in ways that are unattainable in clinical settings, greatly enhancing our understanding and potential for innovation in the field [3]. Mice are widely regarded as excellent models for studying cancer due to their small size, low cost, ease of breeding, ease of genetic engineering, similarity to the human genome, and similar pathogenesis [4]. Various mouse HCC models, encompassing chemical induction, xenografting, and genetic engineering approaches, represent crucial instruments for replicating human HCC’s biological and biochemical characteristics [5]. Recently, an innovative modeling technique utilizing hydrodynamic transfection in conjunction with the Sleeping Beauty transposon system and/or CRISPR/Cas9 has emerged, enabling swift and economical generation of diverse HCC models with various oncogenes or deactivated tumor suppressor genes [6]. This approach addresses the limitations associated with conventional mouse HCC models in transplantation, induction, and transgenic methodologies. The adaptability of these models shows promise in expanding our comprehension of genetic mechanisms underlying human hepatocarcinogenesis, facilitating investigations of both premalignant and malignant liver conditions and assessment of novel therapeutic approaches.

As sequencing techniques advance and become more cost-effective, transcriptomic analysis has emerged as a prevalent tool in exploring HCC pathogenic mechanisms. Nevertheless, transcriptomic studies solely examine HCC pathogenesis through gene functional analysis without considering the regulatory relationships between metabolites, which is insufficient for comprehensively understanding the regulatory network of carcinogenesis. Changes in the metabolite profile may be caused by complex interactions between various environmental factors and gene expression [7]. The integration of transcriptomics and metabolomics may provide more information than any single technique alone, offering deeper pathophysiological insights into tumors [8].

In this study, we sought to identify the signaling pathways changes resulting from transcriptional and metabolic regulation on the carcinogenic malignancy of HCC in hydrodynamically transfected mouse HCC models by metabolomic and transcriptomic analysis and to reveal the underlying biological mechanisms.

## Materials and methods

### Animals

Two independent cohorts of C57BL/6J mice were utilized in this study. SPF C57BL/6J mice were produced by the Experimental Animal Center of Guangxi Medical University. In the survival study, eighteen 7-week-old male mice were randomly allocated into experimental and control groups (n = 9 per group) over a 30-day period. Subsequently, twenty 7-week-old mice (balanced for sex, 10 males/10 females) were stratified by sex and equally allocated to experimental and control groups (n = 10 per group, 5 males/5 females per group). The Experimental Animal Center of Guangxi Medical University provided SPF level feeding environment. The animal investigation was sanctioned by the Institutional Animal Ethical Committee of Guangxi Medical University (No. 202212017).

### Plasmids

pCMV/SB11 was a gift from Perry Hackett (Addgene plasmid #26552; http://n2t.net/addgene:26552; RRID:Addgene_26552) [9]. pT3-EF1a-c-Met (Addgene plasmid #31784; http://n2t.net/addgene:31784; RRID:Addgene_31784) and pT3-N90-beta-catenin (Addgene plasmid #31785; http://n2t.net/addgene:31785; RRID: Addgene_31785) were gifts from Xin Chen [10]. LentiCRISPR-sgP53 and LentiCRISPR-sgPten were constructed using the LentiCRISPR v2 plasmid. LentiCRISPR v2 was a gift from Feng Zhang (Addgene plasmid #52961; http://n2t.net/addgene:52961; RRID:Addgene_52961) [11], and the corresponding sgRNA as follows: P53: CCTCGAGCTCCCTCTGAGCC; Pten: AGATCGTTAGCAGAAACAAA.

### Hydrodynamic injection

The plasmids were extracted from the bacteria by using the endotoxin free plasmid extraction kit (Omega, USA), and the extraction operation was completed under sterile conditions. The plasmid pCMV/SB11, pT3-EF1a-C-Met, pT3-N90-beta-catenin, LentiCRISPR-sgP53, LentiCRISPR-sgPten were combined at a proportion of 1:2:2:2:2, and dissolved in sterile physiological saline, total plasmid concentration was 5.4 μg/mL. The injection dose was 0.1 mL/g according to the body weight of the mice. Mice were arbitrarily split into the experimental group and the control group, and the control group was injected with empty plasmid in the same way and dose.

### Editing effect detection

Extract DNA from a small amount of liver cancer samples. The primers were synthesized by Genesis Biotech. PCR amplification was performed using P53-F: AAAGAGTGCGCCGATAGGTC and P53-R: GCAACAGATCGTCCATGCAG. After purifying, the PCR amplicons were refined and inserted into the pEASY®-T1 Cloning Vector (CT101, TransGen Biotech, Bejing, China). Six colonies were randomly selected and sequenced by Sangon Biotech.

### Serum biochemical indexes analysis and routine blood examination

Whole blood was collected in EDTA-coated collection tubes and blood routine examination were executed by a small-animal routine blood test instrument (URIT, Guilin, China). Non-anticoagulant blood was collected in EP tubes, and serum was procured by centrifugation at 3,000 g for 10 min, the AU5800 series Automatic Biochemical Analyzer (Beckman Kurt, USA). Automatic animal biochemical analyzer 8021AVet (URIT, Guilin, China) was used to obtain serum biochemical analysis data, including alanine aminotransferase (ALT), aspartate aminotransferase (AST), alkaline phosphatase (ALP), total protein (TP), globulin (GLB), albumin (ALB), γ-glutamyl transferase (GGT), total bile acid (TBA), triglyceride (TG), cholesterol (CHOL), and glucose (GLU).

### Detection of pathology

Mice were euthanized and hepatic specimens were preserved in 4% paraformaldehyde solution overnight before paraffin embedding. Tissue blocks were cut into 4 μm thick slices and underwent hematoxylin and eosin (H&E) staining for histological evaluation. For immunohistochemistry analysis (IHC) procedures, the specimens were subjected to antigen recovery under high pressure conditions using citrate buffer solution (pH 6.0) following deparaffinization and hydration steps. IHC was carried out using a Rabbit SP kit (Zhongshan Jinqiao Biotechnology, Bejing, China), antibodies included Anti-Cytokeratin 7 antibody (HUABIO, China), Anti-Cytokeratin 19 antibody (HUABIO, China), Anti-GPC3 antibody (HUABIO, China), Anti-CD34 antibody (HUABIO, China), Anti-PTEN (HUABIO, China), Anti-P53 (Abmart, China), Anti-Met (Abmart, China), Anti-beta Catenin (Abmart, China). The semi-quantitative analysis was performed by using the ImageJ software (NIH).

### Transmission electron microscopy (TEM)

Fresh liver samples were sectioned into 1 mm3 pieces and subsequently immersed in a 3% glutaraldehyde solution for a duration of 2 h. Following this, the samples were rinsed thrice with a 0.1 mol/L PBS solution and then subjected to post-fixation using a 1% osmium tetroxide solution for a period of 1 to 2 h. Thin-section specimens were prepared and then treated with uranyl acetate and lead citrate stains. Ultimately, the specimens were examined with TEM (Hitachi H7650, Tokyo, Japan).

### Metabolome sample preparation

The mouse liver was snap-frozen in liquid nitrogen post-dissection. The frozen tissue was sectioned on dry ice (~ 80 mg) and transferred to an Eppendorf tube (2 mL). The tissue specimens combined with 200 μL of H_2_O and five ceramic beads underwent homogenization utilizing the homogenizer. For metabolite extraction, 800 μL methanol/acetonitrile (1:1, v/v) was introduced to the homogenized mixture. The solution was exposed to centrifugation for 15 min (14,000 g, 4 °C). The resulting supernatant underwent drying in a vacuum centrifuge. Prior to LC–MS analysis, the specimens were reconstituted in 100 μL acetonitrile/water (1:1, v/v) solution.

### LC–MS/MS Analysis

Analysis was performed using an UHPLC (1290 Infinity LC, Agilent Technologies) coupled to a quadrupole time-of-flight (AB Sciex TripleTOF 6600) in Shanghai Applied Protein Technology Co., Ltd.

### RNA Extraction, library sequencing for transcriptome

Total RNA was extracted using Trizol reagent kit (Invitrogen, Carlsbad, CA, USA) according to the manufacturer’s protocol. RNA quality was assessed on an Agilent 2100 Bioanalyzer (Agilent Technologies, Palo Alto, CA, USA) and checked using RNase free agarose gel electrophoresis. The cDNA library was sequenced using Illumina Novaseq6000 by Gene Denovo Biotechnology Co. Ltd. (Guangzhou, China).

### Pathway Enrichment Analysis

The examination of RNA expression differences was executed utilizing DESeq2 [12] software for group comparisons (while edgeR [13] was employed for sample pair analysis). Differentially expressed genes (DEGs)/transcripts were ascertained based on two criteria: false discovery rate (FDR) less than 0.05 and absolute fold change equal to or exceeding 2. Biological functions typically involve multiple genes working in coordination. Understanding gene functions is enhanced through pathway-focused investigations. KEGG [14] serves as the primary public repository for pathway information. Through pathway enrichment analysis, significant metabolic and signal transduction pathways were detected among DEGs when compared against the complete genomic background.

### Analysis of The Cancer Genome Atlas (TCGA) Data

The candidate gene level for LIHC patients, based on their abnormal gene expression, were conducted using UALCAN (http://ualcan.path.uab.edu/). This interactive web resource is designed for the comprehensive analysis of cancer OMICS data, including TCGA and MET500 datasets [15]. The target gene identifier was entered, and the Cancer Genome Atlas database was chosen to assess expression profiles, survival analyses, and associations with associated genes. Default settings were applied for all other parameters.

### Statistical Analysis

The experimental data underwent statistical processing utilizing GraphPad Prism 7.0 (Graph Pad Software, La Jolla, CA, USA). Statistical comparisons between two experimental groups were performed through two-tailed Student’s t test. For statistical evaluation involving three or more experimental groups, one-way analysis of variance (ANOVA) was utilized. Statistical significance was established at *P* ≤ 0.05.

## Result

### Characterization of a hydrodynamic transfection-based HCC mouse model

**Fig. 1:**
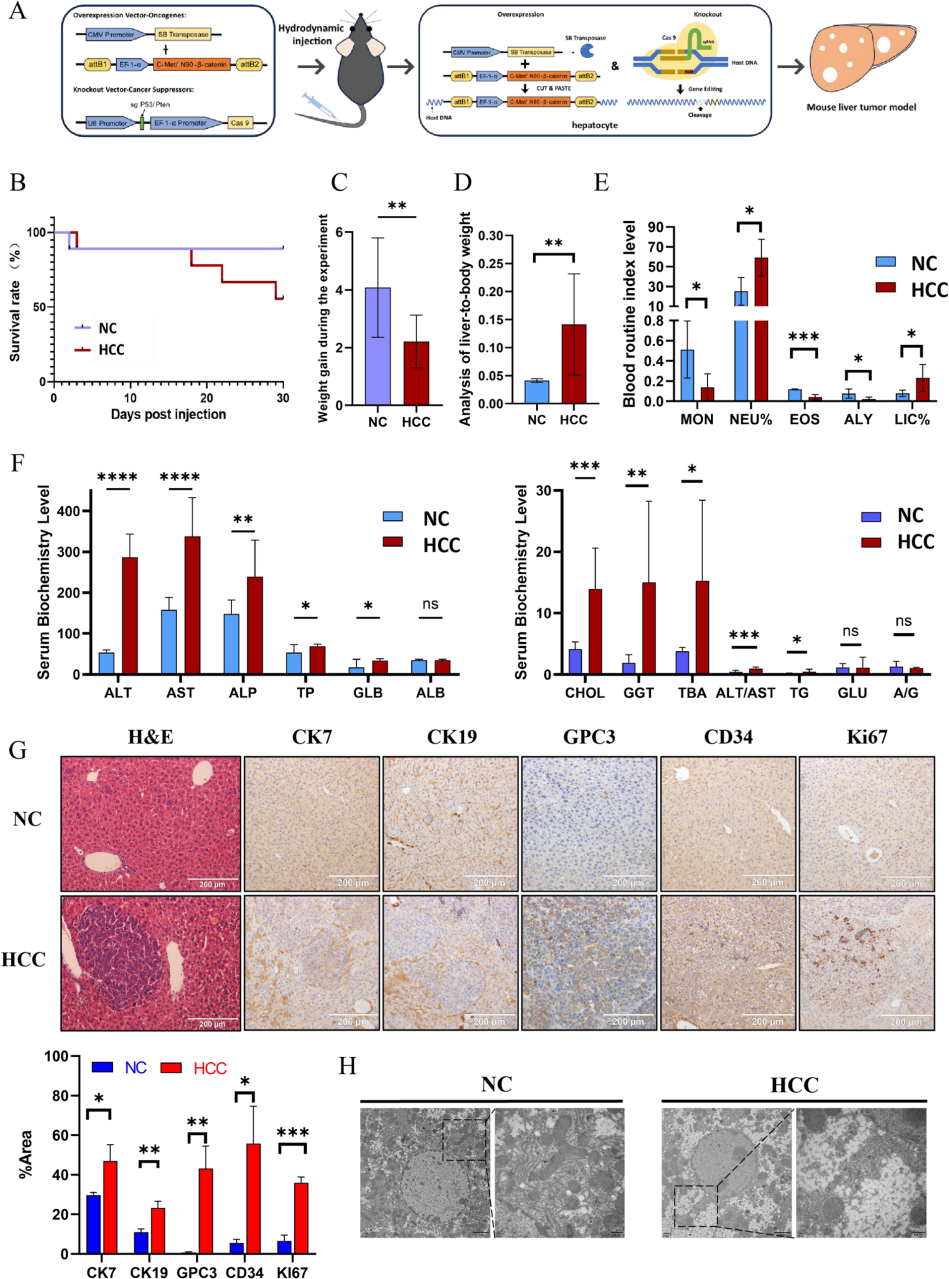
The hydrodynamically transfected mice HCC model was successfully constructed. (**A**) Schematic diagram of hydrodynamic transfection HCC model construction; (**B**) Survival rate analysis of HCC mice model; (**C**) Body weight changes in HCC mice model; (**D**) Liver-to-body weight changes in HCC mice model; (**E**) Routine analysis of blood in the experimental group and control; (**F**) Serum liver function tests suggest that abnormal liver function was induced in the experimental group; (**G**) Histological analysis showed that the liver of the experimental group presented typical pathological features of HCC; (**H**) The liver from the experimental group and control were observed by transmission electron microscopy. NC represents the control group and HCC represents the experimental group. *P < 0.05, **P < 0.01, ***P < 0.001

**Fig. 2:**
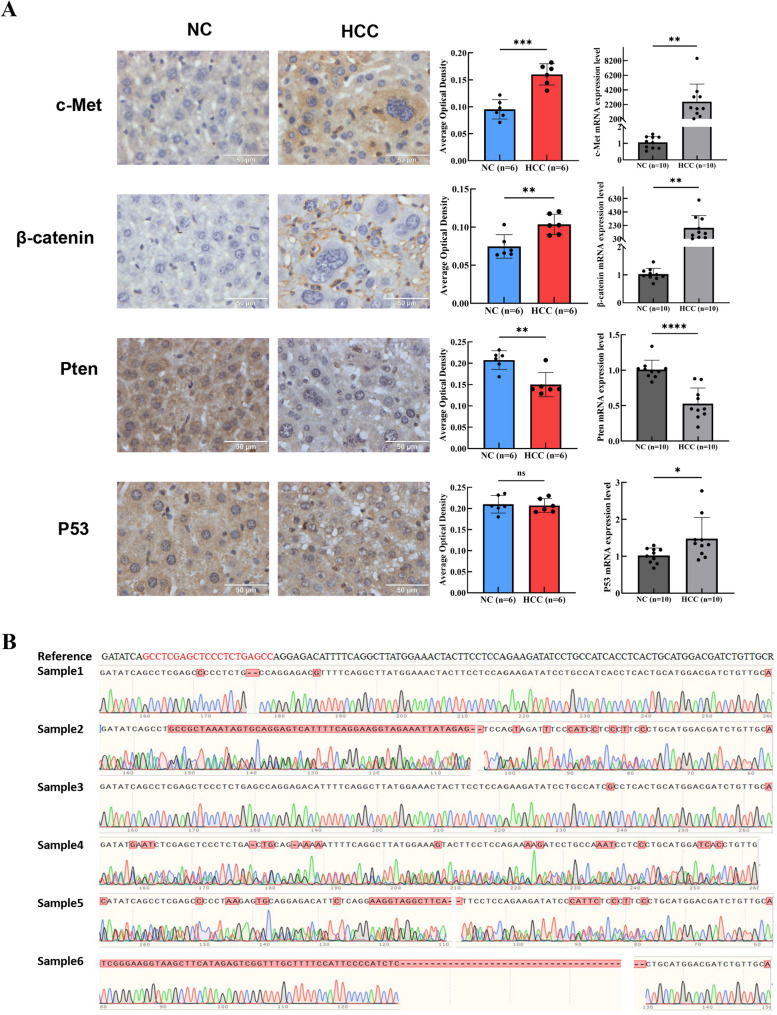
Expression analysis of c-Met, β-catenin, Pten and P53. (**A**) The expression of c-Met, β-catenin, Pten and P53 in liver of experimental group and control group were analyzed by immunohistochemistry; (**B**) Analysis of mutation of *P53* in liver of experimental group by sequencing. NC represents the control group and HCC represents the experimental group. *P < 0.05, **P < 0.01, ***P < 0.001, ****P < 0.0001

A hydrodynamic transfection-based mouse model of HCC was established by hydrodynamic transfection with pCMV/SB11, pT3-EF1a-c-Met, pT3-N90-beta-catenin, LentiCRISPR-sgP53, and LentiCRISPR-sgPten plasmids, following previously reported protocols [16]. This model enables efficient tumor induction in C57BL/6J mice and serves as a reproducible platform for mechanistic studies. The strategy for establishing a mice HCC model is shown in the Fig. 1A. Mortality analysis showed that the mice in the experimental group died gradually from the 18th day after injection due to the aggravation of malignancy (Fig. 1B). Therefore, in our study, both groups of mice were euthanized on the 18th day. It was also found that the experimental group gained much less weight than the control group (Fig. 1C). The liver weight to body weight ratio of the experimental group was markedly higher than that of the control group (Fig. 1D). Blood analysis revealed significant abnormalities in the experimental group versus the control group, including MON (*P* = 0.028), NEU% (*P* = 0.011), EOS (*P* < 0.001), ALY (*P* = 0.040), and LIC% (*P* = 0.035, Fig. 1E). The biochemical indicators of liver function, including ALT (*P* < 0.001), AST (*P* < 0.001), ALT/AST ratio (*P* < 0.001), ALP (*P* = 0.007), GGT (*P* = 0.006), TP (*P* = 0.024), GLB (*P* = 0.019), and TG (*P* = 0.022), were markedly elevated. Additionally, CHOL (*P* < 0.001) and TBA (*P* = 0.012) were also markedly increased compared to the control group. These findings suggest impaired liver function in the experimental group (Fig. 1F). After dissection, the control group’s liver was ruddy and the surface was smooth, with no abnormal conditions, whereas the experimental group’s liver was swollen and congested, with a large number of spherical white masses distributed on the surface and inside, ranging in diameter from 0.5 mm to 10 mm. H&E staining revealed nodules with distinct distribution and varying sizes. The tumor cells were round and oval, with abundant cytoplasm and basophilic lesions, indicating a high degree of malignancy. Nuclear atypia was prominent, with single and multiple nucleoli and mitotic figures (Fig. 1G). Immunohistochemical results demonstrated significant positive expressions of HCC markers CK7, CK19, GPC3, CD34 and Ki67 in the experimental group. Observation of ultrastructure under transmission electron microscopy showed the morphological abnormalities of hepatocyte organelles in the experimental group were pronounced, including nuclear atrophy and malformation, nuclear membrane folding, mitochondrial swelling and vacuolation, and blurring and disorder of organelles such as endoplasmic reticulum (Fig. 1H). The immunohistochemical results indicate active tumor cell proliferation and a high degree of malignancy in the tumor tissue. To verify the expression levels of P53, Pten, c-Met, and β-catenin in mouse liver, immunohistochemical and qRT-PCR analyses were conducted. The results showed a significant increase in the expression of c-Met and β-catenin in the experimental group (*P* < 0.01). Conversely, Pten expression was significantly reduced (*P* < 0.01 in immunohistochemistry, *P* < 0.001 in qRT-PCR), but there was no significant difference in the expression of P53 (Fig. 2A). To further analyze whether P53 in the experimental group underwent gene editing, the P53 sequence was amplified by PCR, and cloned into T vector and transformed into DH5α. The obtained monoclones were sequenced and it was found that all 6 monoclones had DNA mutations in the P53 region (Fig. 2B). The findings indicated that liver cancer in the experimental group of mice is closely linked to mutations in P53 and Pten, as well as the overexpression of c-Met and β-catenin.

### Combined transcriptomics and metabolomics analysis revealed cancer associated pathway enriched in the hydrodynamic transfection model of HCC

**Fig. 3:**
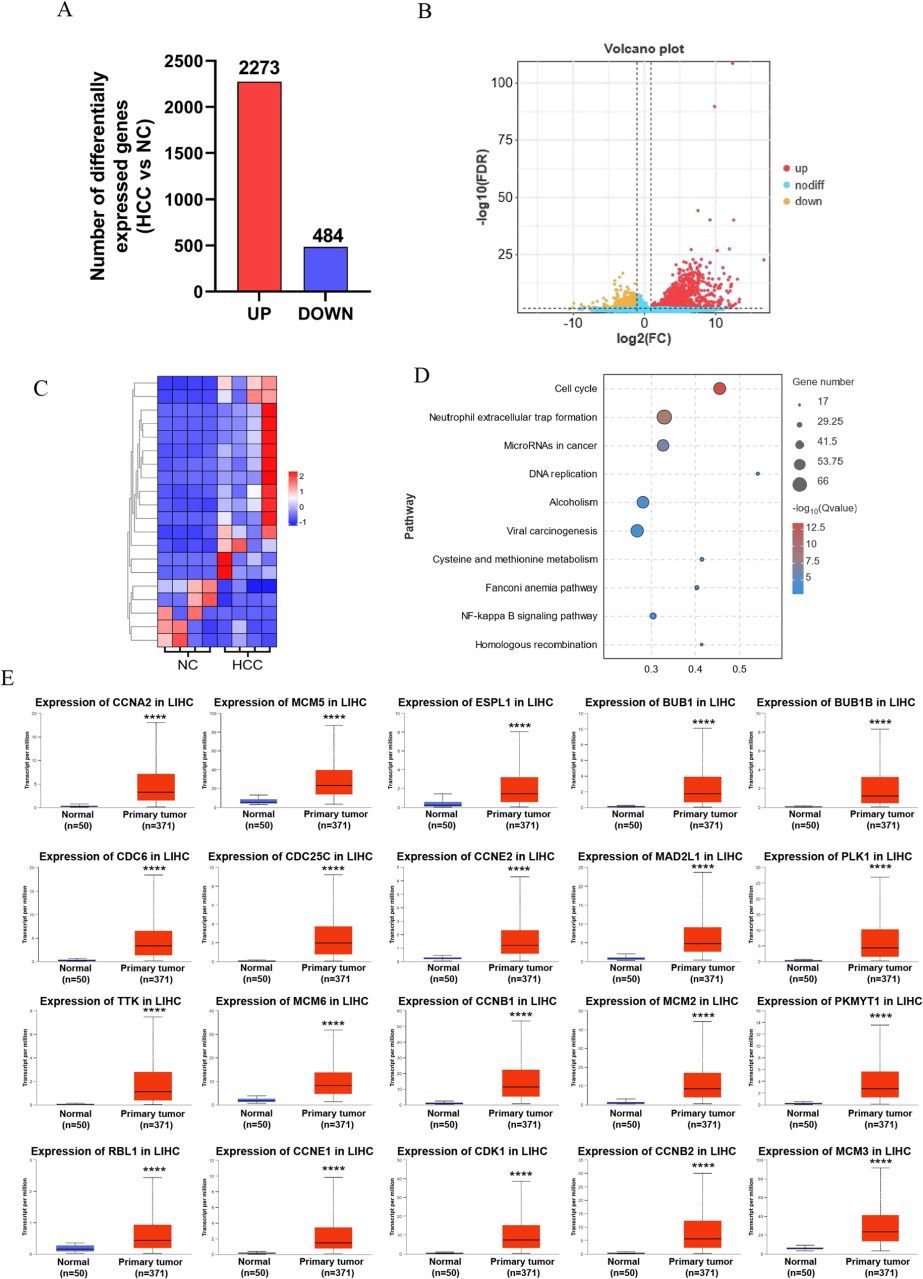
Transcriptomic analysis revealed that differentially expressed genes (DEGs) were significantly enriched into cell cycles pathways. (**A**) Number of upregulated gene and downregulated ones between liver of experimental group and control; (**B**) Volcano plot of gene expression between liver of experimental group and control, Red: high expression, yellow: low expression; (**C**) Top 20 DEGs (|log2FC|> 1, *p* < 0.05) containing upregulated genes (red) and downregulated genes (blue) were visualized by hierarchical clustering based on gene expression difference (log2fold change, *x* axis) and statistical significance (− log10 *p* value, *y* axis); (**D**) KEGG enrichment analysis suggest that DEGs were significantly enriched into cell cycles pathways; (**E**) Analysis of TCGA data showed that expression of Top 20 DEGs was significantly increased in 371 hepatocellular carcinoma (LIHC) patients compared with 50 controls (*P* < 0.0001);

RNA sequencing provides essential understanding for cancer investigation and therapy. As precision medicine continues to advance, RNA-seq has emerged as an effective tool to enhance cancer research exploration [17]. Subsequently, we conducted RNA-seq analysis on liver samples from both experimental and control groups at day 18 following hydrodynamic transfection using the Illumina (HiSeq X-Ten) platform. The transcriptome analysis ascertained 2757 differentially expressed genes (DEGs), among which 2273 exhibited increased expression while 484 showed decreased expression in the experimental group mouse livers (Fig. 3A). Volcanic maps and heat maps are shown in Fig. 3B-C. DEGs and KEGG analysis strongly indicated the enriched pathway were concentrated in cell cycle, neutrophil extracellular trap formation, microRNAs in cancer and DNA replication pathways (Fig. 3D). The cell cycle serves a pivotal function in the progression of liver cancer, and this finding suggests that alterations in the expression of cell cycle-related genes in the experimental group may contribute to the initiation and development of liver cancer. We utilized the TCGA database to analyze the top 20 most markedly differentially expressed genes in the cell cycle pathway between liver cancer tissues from 371 HCC patients and 50 controls. The results indicate that all 20 genes with the most significant differential expression in the cell cycle pathway (*Ccna2*, *Mcm5*, *Espl1*, *Bub1*, *Bub1b*, *Cdc6*, *Cdc25c*, *Ccne2*, *Mad2l1*, *Plk1*, *Ttk*, *Mcm6*, *Ccnb1*, *Mcm2*, *Pkmyt1*, *Rbl1*, *Ccne1*, *Cdk1*, *Ccnb2*, *Mcm3*) are markedly overexpressed in HCC patients (Fig. 3E).

**Fig. 4:**
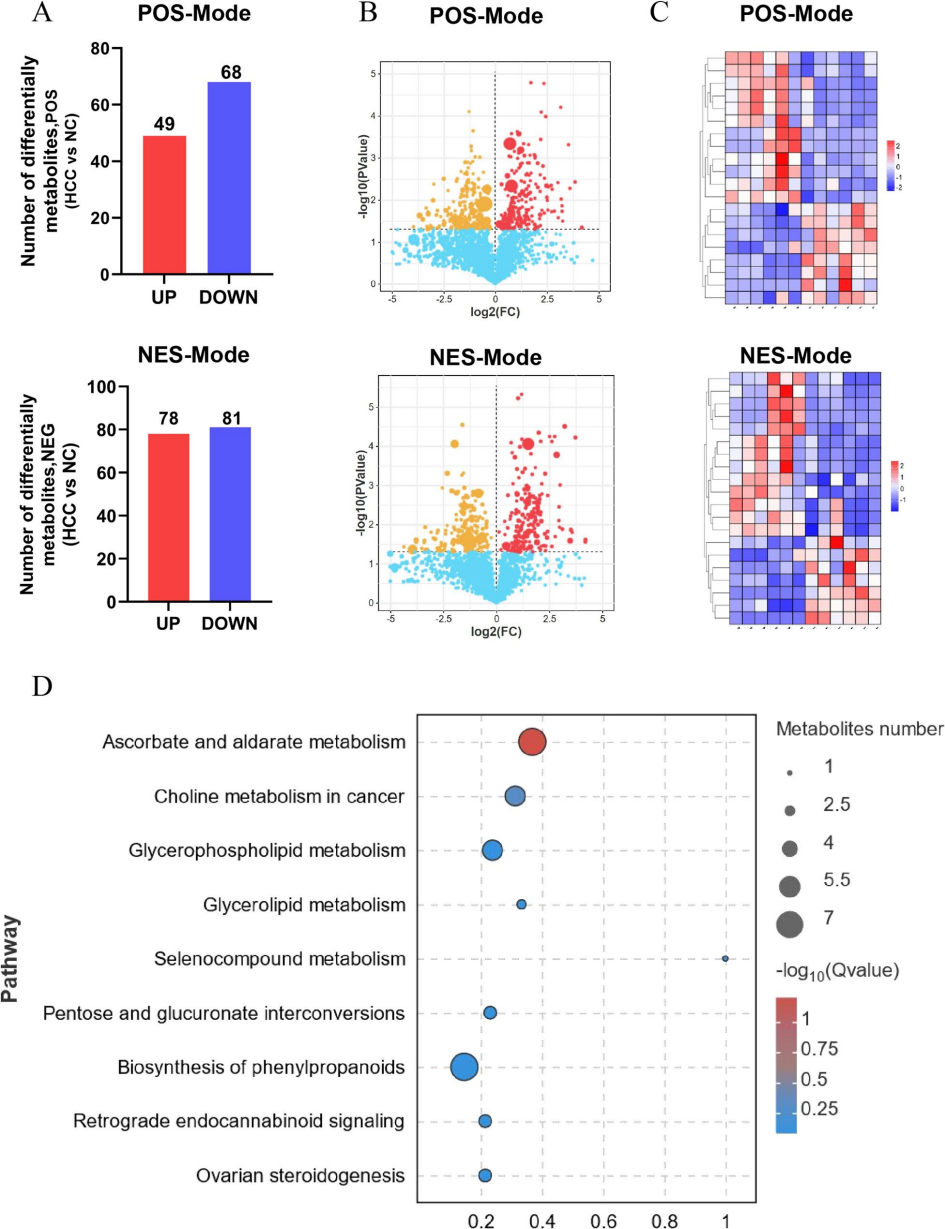
Metabolome analysis revealed that the difference metabolites between the experimental group and the control group. (**A**) Number of upregulated metabolites and downregulated ones between liver of HCC mice group and control; (**B**) Volcano plot of different metabolites between liver of HCC mice group and control, Red: high expression, yellow: low expression; (**C**) Volcano plot of metabolites: displays top 20 significant metabolic alterations between liver of HCC mice group and control. (**D**) KEGG enrichment analysis indicated that the ascorbate and aldarate metabolism pathways significantly enriched with the identified metabolites

Metabolomics analysis showed the expression levels of metabolites in the experimental and control groups’ liver tissues were considerably different. The PCA was performed based on the Paretoscale method. The high degree of clustering of QC samples reflects the examination’s consistency and the trustworthiness of the measurements. In the negative and positive modes, 276 differential metabolites were found, with 127 being upregulated and 149 being down-regulated (Fig. 4A). Volcanic maps and heat maps are shown in Fig. 4B-C. And KEGG pathway enrichment suggested that the differential metabolites were markedly enriched in ascorbate and aldarate metabolism, choline metabolism in cancer glycerophospholipid metabolism, glycerolipid metabolism pathways (Fig. 4D).

### The hydrodynamic transfection model of HCC in mice showed obvious sexual dimorphism

**Fig. 5:**
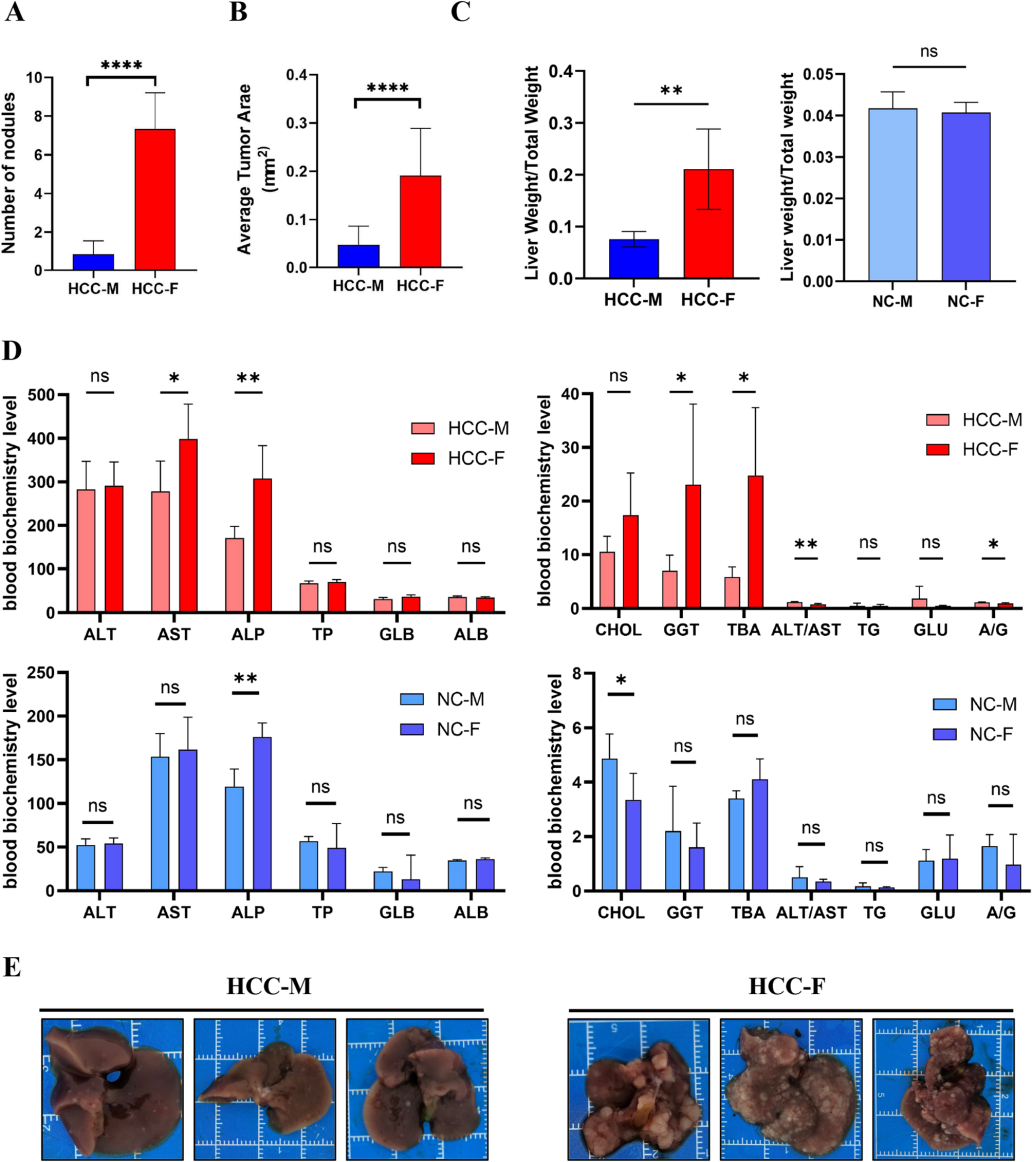
Females enhance malignant progression of HCC in hydrodynamically transfected HCC model. (**A**) Number of nodules analysis of HCC mice model between male and female; (**B**) The average tumor area in female mice was significantly larger than that in male mice in the experimental group; (**C**) The liver weight to body weight ratio of female mice was significantly higher than that of male mice in the experimental group; (**D**) Serum liver function tests suggest that liver dysfunction is more severe in females than male in HCC mice model; (**E**) Photos of livers separated from HCC mice model. HCC-M represents males in the experimental group, and HCC-F represents females in the experimental group. NC-M represents the male in the control group and NC-F represents the female in the control group. *P < 0.05, **P < 0.01, ***P < 0.001, ****P < 0.0001

Notably, we found for the first time a significant difference between males and females in a hydrodynamically transfected HCC model. The hydrodynamic transfection model of HCC in mice revealed a prominent sexual dimorphism, where female mice demonstrated a markedly higher incidence of HCC, heavier livers (*P* < 0.001, Fig. 5A-C). Furthermore, the females in the experimental group exhibited markedly elevated serum levels of AST, GGT and TBA compared to their male counterparts, with no such alterations observed in the control group (Fig. 5D). This elevation in AST levels within the liver suggests a greater degree of liver necrosis in female mice. Additionally, the higher CHOL levels observed in female mice indicate a potential for a more profound impact on lipid metabolism compared to males. To our surprise, it was found that the female livers of the experimental group had more and larger white masses than the male livers (Fig. 5E). All of these data indicate that the incidence and severity of liver tumors in hydrodynamic transfection HCC female mice is much higher than in male mice.

### Integrated analysis of transcriptome and metabolome revealed females enhance HCC by inhibition of glutathione S-transferases (GSTs) in a hydrodynamically transfected HCC mice model

**Fig. 6:**
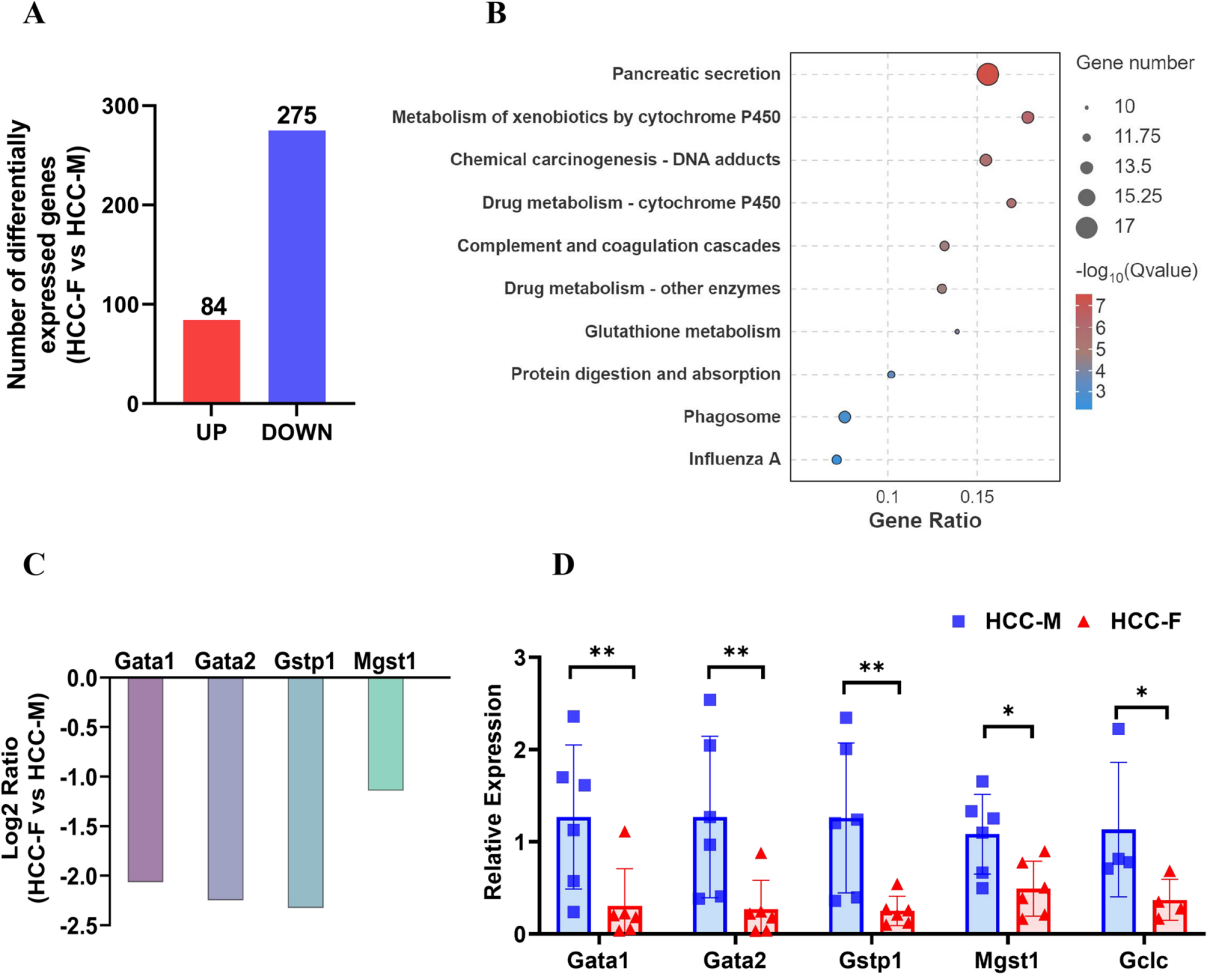
Transcriptomic analysis revealed that differentially expressed genes were significantly enriched into metabolism of xenobiotics by cytochrome P450 pathways. (**A**) Number of upregulated genes and downregulated ones between males and females in liver of HCC mice group; (**B**) KEGG enrichment analysis indicated that differentially expressed genes were significantly enriched into xenobiotics by cytochrome P450 pathways; (**C**) RNA sequencing results of the glutathione transferase family genes. (**D**) qRT-PCR confirmed that low expression of glutathione transferase family genes in females. HCC-M represents males in the experimental group, and HCC-F represents females in the experimental group. *P < 0.05, **P < 0.01

In general, evidence suggests that androgens, and their cognate receptors which contribute to the sex differences in HCC can promote steatosis [18, 19] and tumor initiation and progression [20] whereas the function of estrogens is mainly protective. Our study found, for the first time, that female-induced HCC was more severe than male in our hydrodynamically transfected mouse HCC model. To elucidate the mechanisms involved, we analyzed transcriptional and metabolic differences between females and males in the experimental group. A sum of 359 differentially expressed genes were identified in the transcriptome, of which 84 were upregulated and 275 down-regulated in females (Fig. 6A). The KEGG enrichment outcomes suggested that there are significant differences in the gene expression of pancreatic secretion, cytochrome P450 metabolism, chemical carcinogenesis—DNA adducts, complement and coagulation cascades, and glutathione metabolism between females and males in the experimental group. It is worth noting that multiple genes of the GSTs family were markedly differentially expressed in these enriched pathways (Fig. 6B). Then we validated the result of qRT-PCR was consistent with the transcriptome analysis, which suggest the transcriptome data was a high quality and the expression of GSTs (*Gata1, Gata2, Gstp1, Mgst1*) were markedly lower in female than in male. The expression of *Gclc*, which encodes Glutamate cysteine ligase (Glc), was also notably downregulated in the liver of the female HCC mouse model (Fig. 6C-D).

**Fig. 7:**
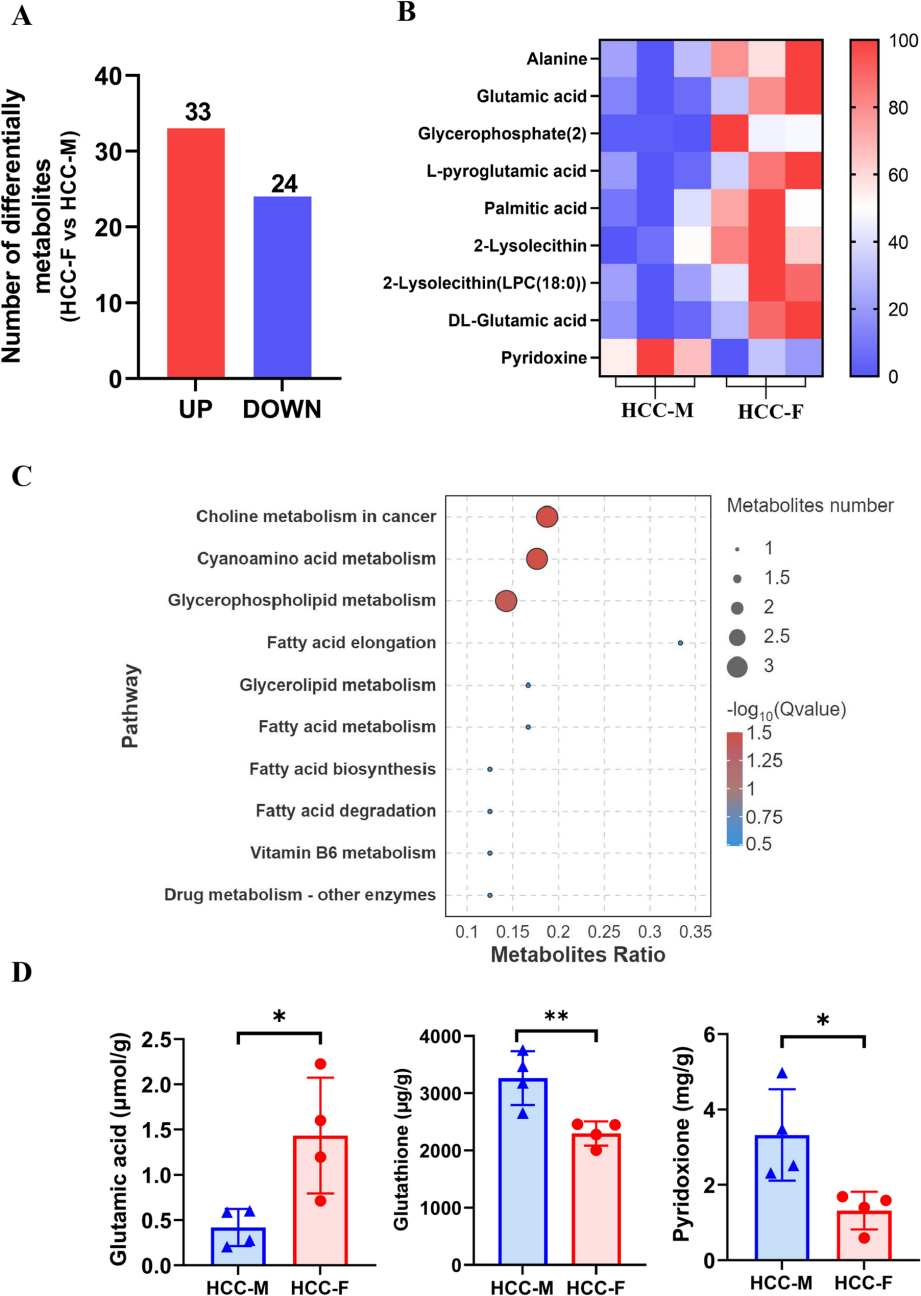
Metabolome analysis revealed that the difference metabolites between the experimental group and the control group. (**A**) Number of upregulated metabolites and downregulated ones between males and females in liver of HCC mice group. (**B**) KEGG enrichment analysis with the identified metabolites; (**C**) Hierarchical clustering of difference metabolites between males and females in liver of HCC mice group, Red: high expression, blue: low expression; (**D**) The content determination of glutamic acid, pyridoxine and glutathione. HCC-M represents males in the experimental group, and HCC-F represents females in the experimental group. *P < 0.05, **P < 0.01

To investigate the metabolite changes between females and males in the experimental group. Totally 57 metabolites (33 upregulated and 24 down-regulated) were detected in females compared with males (Fig. 7A). KEGG pathway enrichment showed that the differentially expressed metabolites enriched in choline metabolism in cancer, cyanoamino acid metabolism, glycerophosphatidylcholine metabolism, fatty acid, glycerolipid metabolism and vitamin B6 metabolism pathways (Fig. 7B). Interestingly, we found several metabolites with significant differences between males and females. glutamic acid, alanine, palmitic acid, lysophosphatidic acid, pyroglutamic acid, and glycerophospholipid were markedly increased in females, while pyridoxine was markedly decreased (Fig. 7C). To validate the metabolism data, the content of glutamic acid and pyridoxine were detected and the result showed that glutamic acid was upregulated and pyridoxine, Glutathione (GSH) were down-regulated in the female HCC mice, consistent with the metabolism analysis (Fig. 7D). Specifically, glutamic acid can be converted to glutathione through a series of biochemical reactions, which is a key step in glutathione synthesis. Glutathione is the most abundant reducing agent in the cell, which has important functions such as anti-oxidation, detoxification and amino acid transport. Integrated analysis of transcriptome and metabolome suggested that low-expression of GSTs can lead to the accumulation of glutathione and glutamic acid, manifested as elevated glutamic acid content.

**Fig. 8:**
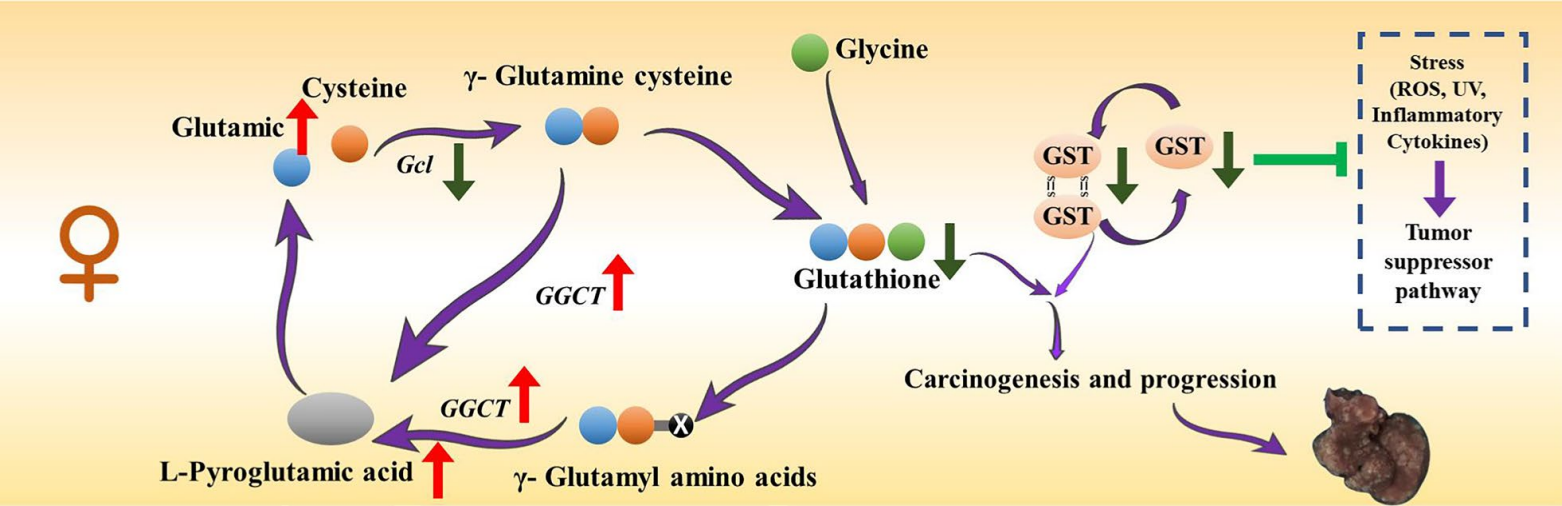
Mechanism diagram on malignant progression of HCC in hydrodynamic transfected HCC mice

In summary, we found for the first time that female mice have a high incidence of HCC, rapid tumor formation and rapid development in a hydrodynamic transfection mice HCC model, which may be due to the decreased expression of GSTs in female HCC mice model, along with the increased content of glutamate, which promotes the malignant progression of HCC (Fig. 8).

## Discussion

Among the experimental models studying liver cancer development, several types exist: NAFLD-linked HCC models, chemical induction approaches, genetically modified mice systems, transgenic and KO specimens, xenograft configurations and patient-sourced xenografts (PDX), which have all aided in elucidating the molecular pathways involved in HCC initiation and advancement [21]. Nevertheless, researchers have not identified a single “ideal” model suitable for all HCC investigative objectives [22]. The hydrodynamic transfection model for HCC in mice involves direct gene editing within the liver of adult mice, resulting in a more effective and significant modeling outcome compared to other methods [23]. Numerous experiments have employed the hydrodynamic transfection technique to construct various HCC models through multi-gene editing, with cancer onset ranging from 4 to 48 weeks [24, 25] Building upon the research of toward, Cao et al. overexpressed c-Met and N90-β-catenin while knocking out P53 and Pten to establish a primary mice HCC model, observing significant tumor development at 6 weeks [10, 16, 26]. In this study, we successfully constructed a mice HCC model using this approach by knocking out the tumor suppressor genes P53 and Pten, and overexpressing the proto-oncogenes c-Met and β-catenin through hydrodynamic transfection. This modeling method is simple, has a high success rate, and closely resembles the development of human primary HCC. Notably, the onset of HCC is markedly faster than reported, occurring within 4 weeks, with a far more severe degree of malignancy. In the experimental group, aggressive HCC development leads to tissue damage and inflammation in the liver. This triggers the immune system to release more neutrophils, a type of white blood cell that responds to tissue injury and inflammation. Additionally, liver cancer can disrupt normal blood cell production (hematopoiesis), further contributing to abnormal blood cell counts. Similar findings have been reported in other studies of liver cancer models, where tumors cause systemic inflammation and blood system changes[27]. This model can be utilized for research on HCC mechanisms and drug screening, laying a foundation for basic research.

In this study, sequencing analysis confirmed mutations in the P53 coding region, likely resulting from non-homologous end joining (NHEJ) following CRISPR-induced DNA double-strand breaks. Interestingly, despite the use of CRISPR/Cas9 to target p53, we observed increased p53 protein levels in tumor tissues. This can be explained by the accumulation of mutant p53 protein, which often exhibits increased stability compared to the wild-type form, leading to detectable levels by IHC despite functional loss. Such mutations are common in human HCC and are associated with poor prognosis. Our findings are consistent with previous reports that TP53 mutations, rather than deletions, are more representative of the clinical context [28, 29]. Rather than achieving complete p53 knockout, our goal was to generate p53 mutations sufficient to initiate tumorigenesis, which reflects the biological scenario in many human HCC cases.

Understanding the signaling pathways that regulate the hepatocarcinogenesis in the hydrodynamic transfection model for HCC is very important for revealing the carcinogenic mechanism of HCC. Combined transcriptomics and metabolomics analysis revealed the enriched pathway was concentrated into in cell cycle, neutrophil extracellular trap formation, microRNAs in cancer and DNA replication pathways in the hydrodynamic transfection model of HCC compared with the control group. The cell cycle represents a series of tightly integrated events that allow the cell to grow and proliferate [30]. Abnormal cell cycle progression is one of the basic mechanisms of tumorigenesis [31]. It has been reported that in addition to well-known innate immune functions, neutrophil extracellular traps (NETs) are also essential players in different phases of tumor initiation and advancement. They enhance tumor angiogenesis and development, support tumor expansion, and create a defensive barrier for tumor cells against anti-tumor immunity [32]. The transcriptome data enriched cancer-related pathways, which provided a reliable basis for the study of the carcinogenic mechanism of HCC by hydrodynamic transfection in HCC mice model.

Studies worldwide indicate that HCC occurrence and frequency remain elevated among men compared to pre-menopausal women, pointing toward potential gender-based differences influencing liver disease development and advancement [33]. Our study found that female-induced HCC in our hydrodynamic transfected mice HCC model was more severe than in males. In humans, HCC often results from the interaction between genetic factors and environmental carcinogens. However, in our model, HCC is mainly driven by genetic alterations without exposure to environmental carcinogens, which are important in human HCC. In addition, there are species differences between mice and humans that may influence tumor development. Our findings reflect real and consistent results from our experiments. This also highlights the need for researchers using this model to consider potential sex-related differences. Sex-related differences have also been observed in other tumor models. Research shows that female rodents exhibit markedly reduced rates of both spontaneous and carcinogen-triggered liver tumors compared to their male counterparts [34]. Experimental NAFLD studies demonstrate accelerated tumor development in male subjects, while females show decelerated progression, particularly when comparing lean versus obese NASH-HCC cases [35]. Within chemical and diet-based experimental models, HCC manifests in all male subjects but only one-third of females, accompanied by substantial chromosomal alterations [36]. In genetic models, 100% HCC present in males and of 30% in females Alb-c-myc/MT-TGF-a mice [37]. Transgenic mice overexpressing miR-221 in the liver present a spontaneous HCC development in 50% of male mice, no liver tumors in females [38]. Integrated analysis of transcriptome and metabolome revealed females enhance HCC by inhibition of GSTs. GSTs (EC 2.5.1.18) represent a category of phase II detoxifying enzymes that facilitate the binding of glutathione (GSH) to various chemical compounds with diverse structures and functions, generating the respective GSH-linked products [39]. p53 controls GSTP1 gene expression and safeguards genetic material from alkylating agents and molecules that generate free radicals. Within cancerous cells, p53-mediated GSTP1 gene activation enhances cell survival and chemoresistance, while in healthy cells this mechanism provides defense against genotoxic substances [40]. Previous research has indicated that tissues exhibiting diminished GSH or GST levels might show a decreased ability to neutralize carcinogenic compounds, potentially resulting in enhanced cytogenetic injury, which could subsequently increase cancer susceptibility [41]. The liver function indexes and electron microscopy images of the two groups of mice in this experiment showed that the hepatocytes in the experimental group were seriously damaged. Liver cell damage is not just a background factor-it is a key feature of disease progression. The metabolomic changes we observed likely reflect tumor-related metabolic reprogramming.

In this study, the expression of GSTs (Gata1, Gata2, Gstp1, Mgst1) was markedly reduced in the female experimental group. Previous studies have shown that glutathione S-transferase (GST) expression in the liver is influenced by sex-related hormonal regulation. A classic study by Srivastava et al. reported that the expression of several hepatic GST genes is lower in females due to differences in growth hormone (GH) secretion patterns between sexes[42]. Specifically, the more continuous GH secretion in females leads to reduced activation of GST gene transcription, which may explain the decreased GST expression observed in female mice in our model. In addition, estrogen has been shown to suppress the expression of detoxification enzymes, including GSTs, further contributing to sex-based differences in glutathione metabolism[43]. Recent research also suggests that sex-specific epigenetic modifications, such as DNA methylation, can stably influence GST gene expression[44]. These findings support the idea that lower GST expression in female mice is likely caused by the combined effects of GH signaling, estrogen regulation, and epigenetic control. These factors may contribute to the increased vulnerability to HCC in female mice. In the GSTs, GSTP1 is an enzyme implicated in the detoxification of carcinogenic electrophiles. Polymorphism at codon 105 in exon 5 and codon 114 in exon 6 of GSTP1 results in reduced enzymatic detoxification activity, which contributes to the development of many cancers [45]. Overexpression of GSTP1 inhibits the proliferation of HepG2 and Huh7 HCC cells both in vivo and in vitro. GSTP1 arrests the cell cycle at G1/S by upregulating p21 and p27 and downregulating p-Akt. Blocking the expression of the GSTP1 gene promotes the proliferation of HCC cells and increases the proportion of cells in the S phase by reducing the levels of p21 and p27 and increasing the level of p-Akt. These results indicate that high levels of GSTP1 provide a better prognosis by inhibiting tumorigenesis in HCC [46]. Analysis of differentially metabolized metabolites between males and females in the experimental group revealed markedly increased levels of glutamic acid, alanine, palmitic acid, lysophosphatidic acid, pyroglutamic acid, and glycerophospholipids in females, while the level of pyridoxine was markedly decreased. Recent studies reveal that glutamine synthetase (GS) inhibition elevates circulating glutamate levels, which paradoxically accelerates liver regeneration while potentially fueling HCC growth [47]. However, the relationship between liver regeneration and HCC progression is complex. While regeneration can repair liver tissue, it may also create an environment conducive to tumor growth. Studies have highlighted the role of growth factors in both liver carcinogenesis and regeneration, emphasizing the need to balance promoting regeneration and preventing cancer recurrence [48]. Increased glutamate levels may accelerate liver regeneration, their impact on HCC progression requires further investigation to fully understand these complex interactions. Multi-omics integrated analysis provides important clues and evidence for a deeper understanding of gene regulation and metabolic networks in organisms within liver cancer models, revealing the mechanisms underlying the occurrence and development of liver cancer and identifying potential therapeutic targets.

Combining all, we propose that the low expression of GSTs contributes to the exacerbation of malignant progression of HCC in female hydrodynamic transfected HCC mice compared to male mice. However, a notable limitation of our study is the lack of mechanistic insights explaining why the observed sex difference in our model contrasts with findings from other models. Another limitation of this study is the small sample size. While we observed that HCC was more aggressive in female mice—opposite to previous reports—this result should be interpreted with caution. Future studies with larger samples are encouraged for further validation. Although our findings suggest that reduced GST expression and altered glutamate metabolism may contribute to more aggressive HCC in female mice, further in vitro and in vivo experiments using GST knockdown are needed to clarify its role in HCC development and glutamate metabolism, which may help identify novel therapeutic targets or strategies.

## Data Availability

No datasets were generated or analysed during the current study.
